# Genome analyses revealed genetic admixture and selection signatures in *Bos indicus*

**DOI:** 10.1038/s41598-021-01144-2

**Published:** 2021-11-09

**Authors:** S. P. Dixit, A. K. Bhatia, Indrajit Ganguly, Sanjeev Singh, Soumya Dash, Anurodh Sharma, N. Anandkumar, A. K. Dang, S. Jayakumar

**Affiliations:** 1 ICAR - National Bureau of Animal Genetic Resources, Karnal, Haryana 132001 India; 2 ICAR - National Dairy Research Institute, Karnal, Haryana, 132001 India

**Keywords:** Evolution, Genetics

## Abstract

The genomic diversity and relationship among seven diverse cattle breeds viz. Sahiwal, Tharparkar, Gir, Vechur, Ongole, Kangayam and Hariana were investigated in 132 random samples based on high density SNP array comprising > 777 K SNPs. A total of 1993 SNPs (0.25% of the total) having greater power (F_ST_ ≥ 0.20) to differentiate these cattle populations were identified, and utilized to partition genome of each animal into a predefined number of clusters. The structure of these cattle indicated shared ancestry of dairy breeds viz. Gir, Tharparkar and Sahiwal. Most of the animals (> 76%) of different populations under study except Vechur clustered into their own group of animals called breed. Vechur population retained highest rate of admixture, consistent with its crossing with other breeds. Ongole, Kangayam and Hariana shared comparatively less of their genome (≤ 15%) with other breeds. The study indicated that all seven breeds evolved from their independent ancestry but there was intermixing of these breeds in the recent past. The selection signatures identified between draft (Kangayam) and dairy breeds included several genes like *FAM19A2, RAB31P, BEST3, DGKA, AHCY, PIGU* and *PFKP* which are involved in immune response, metabolic pathway, transportation of glucose and sugars, signaling pathways, cellular processes, cell division and glycolysis regulation, respectively. Moreover, these genomic regions also harbour QTLs affecting milk performance traits. The signatures were also identified even between the dairy breeds. In comparison to large-sized cattle, there were significant differences in the number of QTLs affecting production (body weight, growth rate etc.) and morphological traits (height) in short-statured Vechur breed. The presence of *HMGA2* gene in the selection signature on chromosome 5 may explain the variations in stature between these cattle.

## Introduction

India has 50 registered cattle breeds adapted to different agro-climatic conditions, breeding and management practices (https://nbagr.icar.gov.in/en/registered-cattle/) and are likely to differ for a number of traits. These breeds thrive in humid subtropical, semi-arid & arid and tropical wet/dry climatic regions, catering to a variety of specialized functions such as dairy, draft and dual (Dairy & draft) use. Therefore, these cattle would have gene content unique to such roles and adaptations in their genome^1^. Hence, they can serve as a great reservoir of genetic pool for identifying genes under selection for different traits that have evolved, as well as for determining genetic diversity.

In India, cattle account for approximately 58% of the country's 303.76 million bovines, but share only around 48% of the total milk produced, due to their low dairy productivity and a higher proportion of draught and dual-purpose breeds than dairy breeds^2^. With the exception of Africa, where output remained stable, global milk production increased 2.0% from 2019 to nearly 906 million tonnes in 2020, owing to rises in all geographical regions. Milk production in India reached to 195 million tonnes in 2020, up 2.0% from the previous year^3^ and accounting for around 21% of global milk production. In addition to their dairy usefulness, draft/dual cattle breeds in India are also used for agricultural and transport operations. Considering their importance in Indian agriculture, several government schemes, such as National Programme for Bovine Breeding and Dairy Development, National Dairy Plan and Dairy Entrepreneurship Development scheme have been launched to boost their future productivity.

The use of single nucleotide polymorphism (SNP) array and next-generation sequencing technologies in genomics and population genetics has resulted significant progress in deciphering the genetic structures^4^, genome diversity and selection footprints in cattle^1,5–11^. Understanding the genomic diversity of native cattle breeds aids in improving their productivity, fitness, fertility and even the behaviour. Additionally, using high-density SNP arrays can increase detection limits of positive selection and lower the false discovery rate^12–15^. Only a few reports using Illumina high-density SNP arrays on Indian native cattle breeds are available^1,16,17^.

To better understand the genetic mechanisms underlying the local adaptation and functional characteristics of Indian zebu cattle, we investigated three dairy breeds (Sahiwal, Gir, Tharparkar) from sub-tropical and hot arid regions, two dual breeds (Hariana, Ongole) from sub-tropical and hot humid regions, and one draught breed (Kangayam) from the country's hot humid region. We have also included Vechur, a short statured cattle breed native to hot humid climate. This array of cattle breeds, each adapted to a different ecological niche and functional attribute, will help to uncover genomic diversity and natural and artificial selection footprints over centuries. Previously, we identified and characterized genome wide runs of homozygosity (ROH) signatures in these cattle breeds using Illumina BovineHD BeadChip^17^. The objectives of the present study were to: (1) assess genomic diversity and effective population size; (2) analyse admixture and structuring; (3) identify diversified selection signatures among breeds using SNP data.

## Results and discussion

### Genomic diversity within and among the breeds

Twenty of the 132 animals were excluded due to low genotyping (MIND > 0.1), and the average genotyping rate for the remaining 112 animals was 0.99. The final data on 112 cattle samples belonging to Sahiwal (13), Tharparkar (17), Gir (15), Ongole (17), Hariana (18), Kangayam (16) and Vechur (16) breeds were achieved after quality control measures (Table 1). A sample size of ≥ 13 per breed was adequate for the diversity analyses, which was in consonance with other studies^18,19^. Out of 735,293 autosomal SNPs genotyped in these cattle, 60% (438,176) were in Hardy Weinberg equilibrium and revealed higher degree of polymorphism (MAF > 0.05). It has also been suggested that if the number of markers is large enough, a sample size of 4–6^20^ and polymorphic SNP filtration^17,19,21^ could offset the influence of ascertainment bias, as in the present work. The SNPs in strong LD (R = 0.5) were also filtered out to minimize the bias in estimating the genomic diversity. We were left with 165,021 informative SNPs after pruning for further genome diversity analyses. Minor allele frequency varied between breeds, ranging from 0.23 (Kangayam) to 0.26 (Vechur), with an average observed heterozygosity of 0.35 in all samples examined (Table 1) and these values were also reported earlier in these breeds^17^. The similar estimates of MAF have been recorded in Nellore, Holstein, Iranian, Ethiopian and South African cattle^22–26^, which varied from 0.21 to 0.25 in *Bos indicus* and *Bos taurus* cattle breeds. However, higher/lower estimates were also observed in other cattle breeds^27–29^. The observed heterozygosity in several zebu cattle^25,30,31^ were in agreement with the present estimates. However, higher heterozygosity have been reported in Hanwoo, Rwanda and other Taurine cattle breeds^25,29,30^. The lower genetic variability estimates in *Bos indicus* relative to *Bos taurus* were consistent with earlier studies^29,32,33^. Out of Bovine HD and 54 K SNP chips of Illumina, only 40–50% SNPs were found to be informative for genetic diversity of Zebu cattle breeds of India^16^ but, it was 90% in *Bos taurus* after quality control^14^. As a result, a *Bos indicus*-specific SNP chip could be more informative because it can capture diversity at nearly all of the loci tiled in the array.

**Table 1:** Number of animals, mean of expected and observed heterozygosity (He, Ho), minor allele frequency (MAF) and coefficient of inbreeding (F_IS_).

Breed	n	He	Ho	MAF	F_IS_ (*p* > 0.40)
Sahiwal	13	0.34	0.35	0.25	− 0.009
Tharparkar	17	0.33	0.34	0.24	− 0.003
Gir	15	0.32	0.33	0.24	0.010
Ongole	17	0.33	0.34	0.24	0.006
Hariana	18	0.33	0.34	0.25	− 0.001
Kangayam	16	0.30	0.33	0.23	− 0.062
Vechur	16	0.34	0.35	0.26	− 0.009
Overall	112	0.36	0.35	0.27	− 0.009

Initially, all filtered SNPs (4 38,176) were utilized to determine the genome diversity of seven cattle breeds: Tharparkar, Sahiwal, Gir, Vechur, Ongole, Kangayam and Hariana. After that, only differentiating loci with greater power to distinguish these cattle populations (F_ST_ ≥ 0.20) were chosen for assessing genetic diversity, selection signature and breed structuring. Hence, out of 777 K, 1993 loci (0.25% of the total) were used for further analysis. The genetic differentiation (F_ST_) of the breeds based on all the SNPs was just 0.05 and based on 1993 most differentiating loci was 0.23. The gene diversity among breeds (Dst), and Dest, a measure of population differentiation^34^, across the loci were 0.08 and 0.13, respectively.

**Figure 1:**
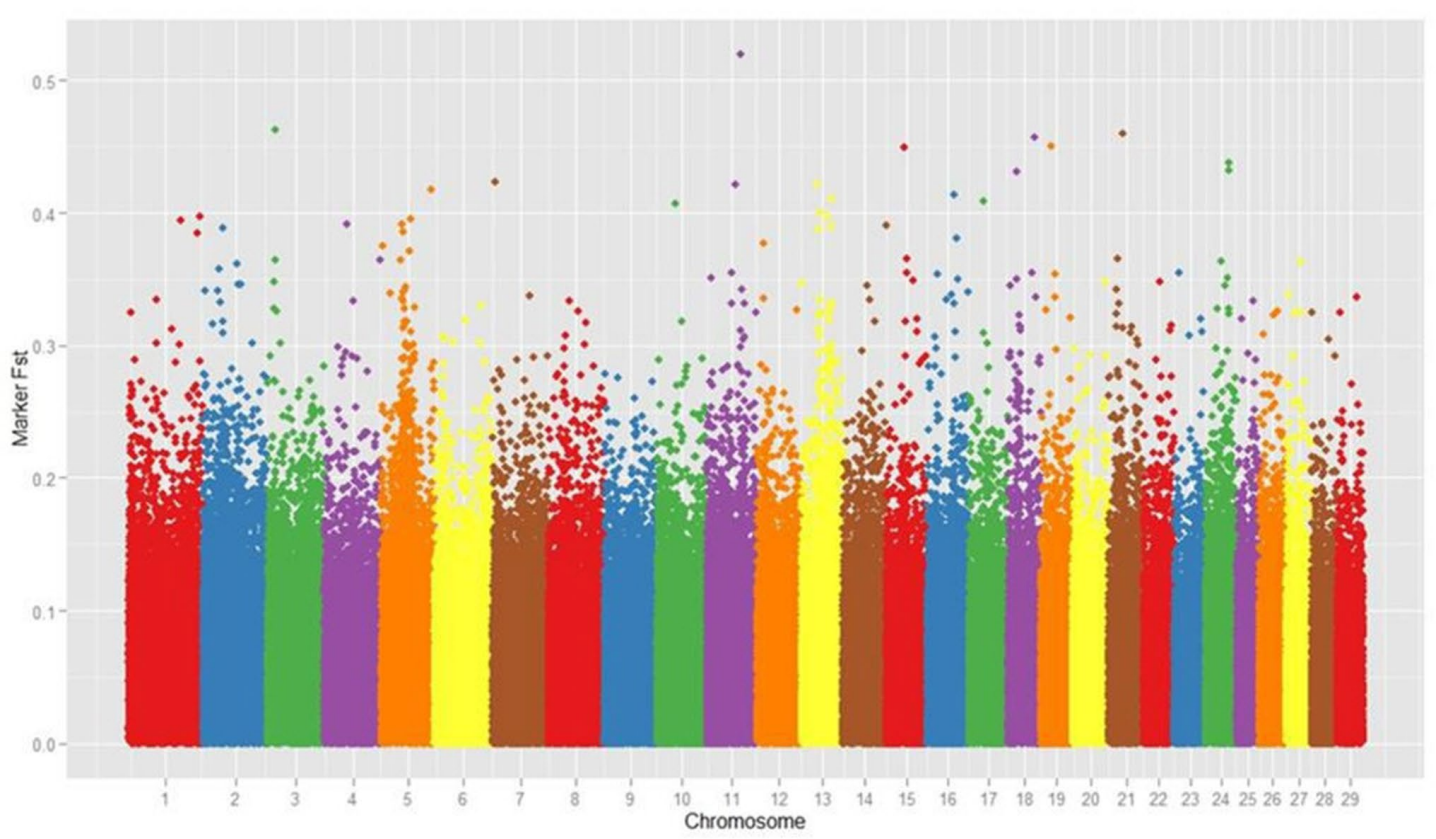
Distribution of marker F_ST_ values across the chromosomes using Manhattan plot.

**Figure 2:**
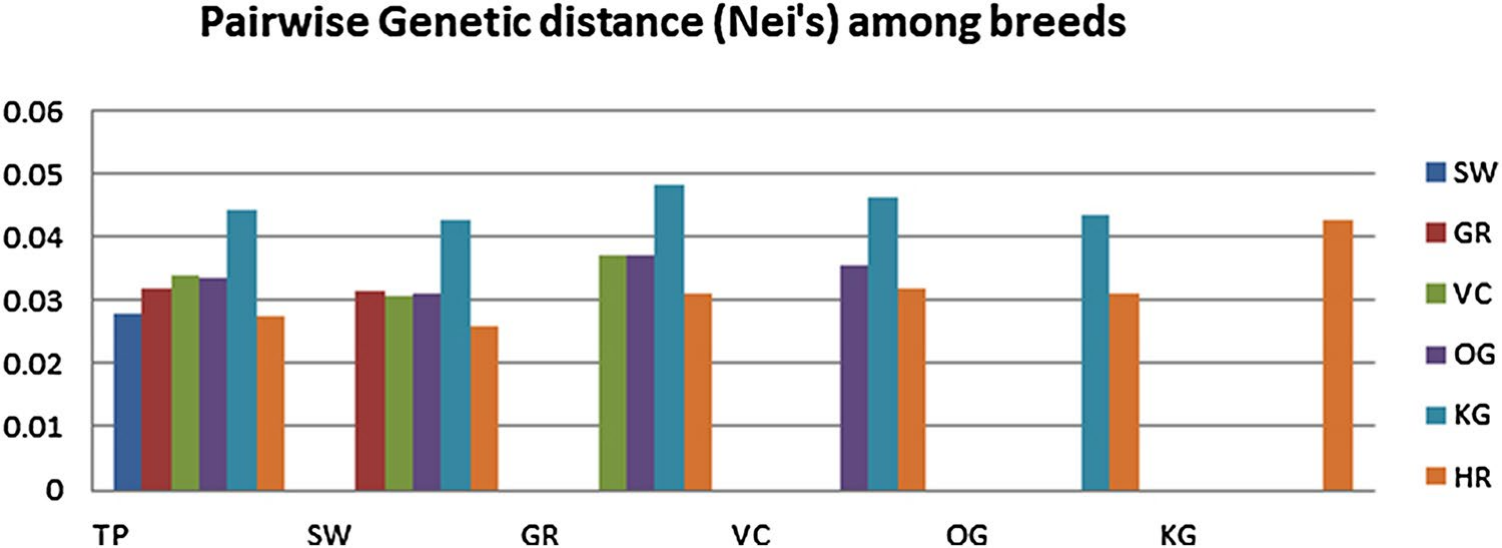
Pair-wise Genetic distance (Nei’s) among breeds. The abbreviations are defined below: TP—Tharparkar, SW—Sahiwal, GR—Gir, VC—Vechur, OG—Ongole, KG—Kangayam, HR—Hariana.

Manhattan graph (Fig. 1) depicts the distribution of F_ST_ values across the chromosomes. The spread of F_ST_ showed that there are few loci (16 markers) having the higher degree of genetic differentiation (F_ST_ ≥ 0.40). The inbreeding coefficient (identical by state) was zero, suggesting that the cattle from which the samples were taken were randomly mated. The genetic differentiation power of those informative SNP loci (n = 1,993) in cattle breeds under study ranged from 0.20 to 0.51, with an average value of 0.23, indicating that these loci account for 23% of the genetic variation between the breeds. Table 2 and Fig. 2 showed the pair-wise estimates of F-statistics (F_ST_) and Nei's genetic distance^35^. Both estimates revealed higher genetic differentiation between Kangayam and rest of the cattle breeds (F_ST_: 0.08 to 0.10), followed by Ongole and rest of the breeds (F_ST_: 0.05 to 0.07). Dairy cattle breeds viz., Tharparkar, Sahiwal and Gir showed the least genetic differentiation (F_ST_: 0.04 to 0.06). A moderate genetic differentiation^36^ where F_ST_ ranged from 0.05 to 0.15 have been recorded in *Bos taurus* dairy and beef cattle with an F_ST_ value of 0.08 across all SNPs^14^, as well as in African cattle breeds (F_ST_ : 0.04 to 0.08). Many SNPs with higher genetic differentiation power (F_ST_ > 0.5) have also been reported in *Bos taurus* dairy and beef cattle^14^. The degree of genetic differentiation among Indian Zebu cattle was comparable to that of African Zebu cattle^30^, but lower than that of *Bos taurus* and *Bos indicus*^30,33^, as anticipated given their historical divergence.

**Table 2:** The pair-wise Nei's F_ST_ (above diagonal) and genetic distance (below diagonal) among the cattle breeds.

Breed	TP	SW	GR	VC	OG	KG	HR
TP	–	0.044	0.059	0.066	0.064	0.094	0.049
SW	0.028	–	0.051	0.051	0.052	0.081	0.037
GR	0.032	0.032	–	0.072	0.072	0.101	0.056
VC	0.034	0.031	0.037	–	0.069	0.096	0.061
OG	0.033	0.031	0.037	0.035	–	0.092	0.058
KG	0.044	0.042	0.048	0.046	0.043	–	0.088
HR	0.027	0.026	0.031	0.032	0.031	0.043	–

SW-Sahiwal, TP-Tharparkar, GR-Gir, VC-Vechur, OG-Ongole, KG-Kangayam, HR-Hariana.

### Effective population size

**Figure 3:**
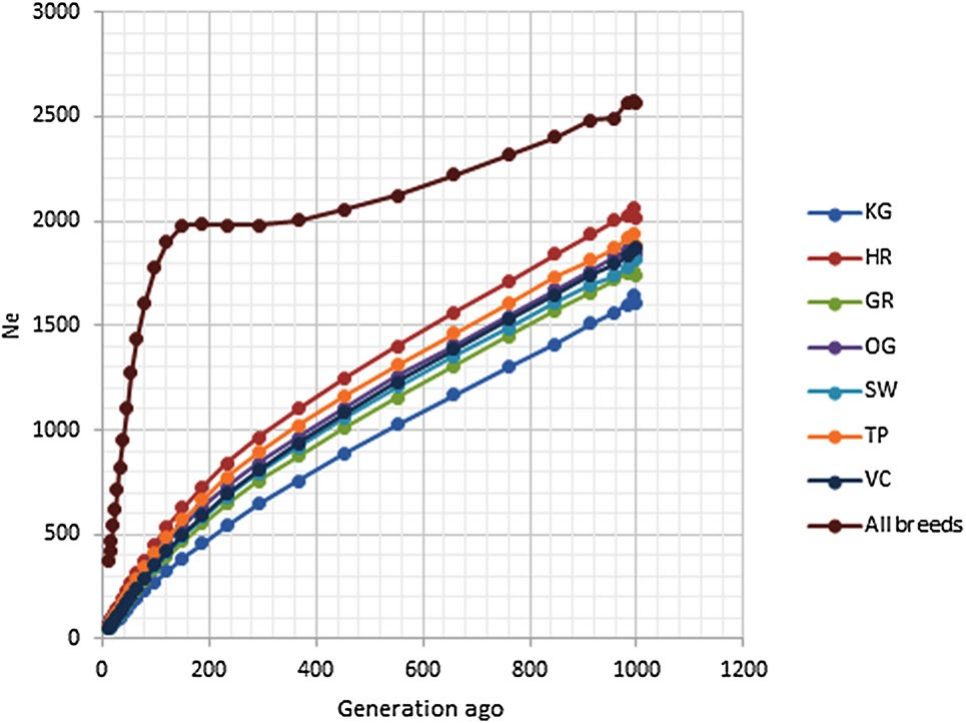
Effective population size (*Ne*) of Indian cattle breeds for a number of generations. X and Y axis represents generation and *Ne,* respectively.

Ancestral and recent effective population sizes (*Ne*) for seven Indian cattle breeds are presented in Fig. 3. Estimated *Ne* showed a downward trend in recent generations across the populations. The most rapidly declining recent Ne was found in the KG and GR, whereas HR and TP showed a slowly declining trend. The estimated *Ne* at 13 generations ago in seven Indian native cattle breeds ranged from 48 to 74 (Supplementary Table S1).

Over the last 999 years, the *Ne* has displayed a decreasing pattern across Indian breeds, with a steeper slope since about 200 generations ago. Lower *Ne* had been found in KG and SW with estimated values of 48 and 51, respectively, 13 generations ago (*Ne*_*13*_), due to the intensive selection pressure or artificial insemination used for developing these breeds. In Kangayam cattle, we have observed maximum autozygosity attributable to both recent and ancient inbreeding^17^.

Recently, a Bovine HD-SNPs array based screening of Chinese native cattle populations revealed a similar trend, with *Ne*_*13*_ values ranging from 85 to 132^37^. The genotyping of Italian local beef breeds (Calvana-CAL, Mucca Pisana-MUP, Pontremolese-PON) and Italian Limousin (LIM) using the GeneSeek GGP-LDv4 33 k SNP chip containing 30,111 SNPs showed an average estimated historical effective population size (*Ne*_*13*_) of 45–310 (CAL-79, MUP-65, PON-45 and LIM-310)^38^.The application of LD-based *Ne* estimation in developing countries for local breeds without pedigree information could offer new perspectives for the assessment of the actual gene pool available and the respective decision-making in conservation and management.

### Genetic structuring and classification of the cattle breeds

**Figure 4:**
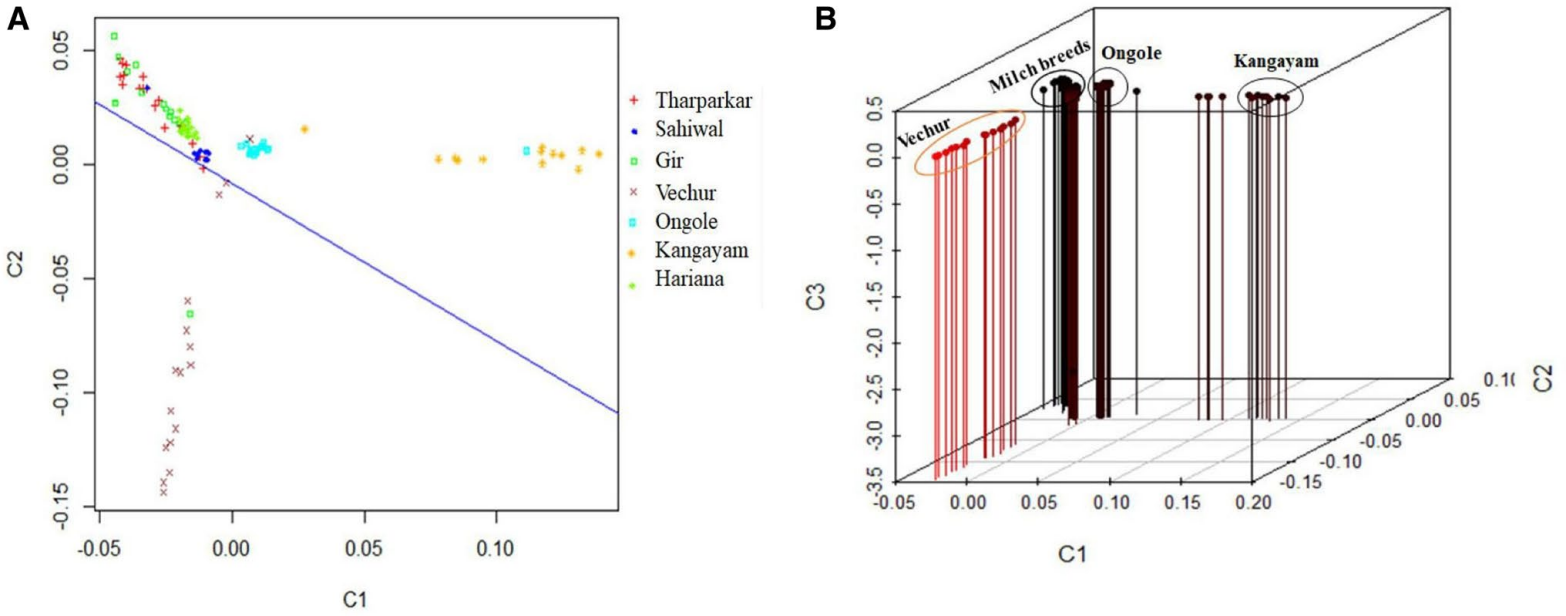
Multi-dimensional scaling plots of genome-wide IBS pairwise distances. A: 2D plot, wherein blue line represents the rotated X-axis (PC1 space) and explains maximum variability of the data; B: 3D plot.

The principal component analysis (PCA) based on genomic relationship (IBS-Identity by state) matrix using 165,021 LD pruned autosomal SNPs was undertaken to assess breed composition of the animals. The first, second and third principal components accounted for 28.7, 11.9 and 6.8% of the total variation, respectively. However, the first three components based on runs of homozygosity explained 98.7% cumulative variation in these breeds^17^. Figure 4 presented the first, second and third principal components where Vechur, Kangayam and Ongole clustered separately from other breeds under investigation, and rest of the breeds (Gir, Sahiwal, Tharparkar and Hariana) grouped together. The structure and PCA both revealed that dairy breeds clustered apart from the dual (Ongole) except Hariana, and draft breeds. PCA based on SNP data clearly separated the breeds by their utility and size, which was in consonance with analysis of molecular variation (Table 3). Kangayam, a draft breed and Vechur, a small statured breed were quite distinct from the dairy and dual breeds and was in agreement with structuring of these cattle based on runs of homozygosity (ROH)^17^. Moreover, the structure and genomic relationship among these cattle was also studied using a small number of highly differentiated loci (180, 50, 10 markers) and it was interesting to note that even 10 markers also revealed the same level of differentiation as with large sized battery of markers. Therefore, the structure and relationship between the cattle breeds were not significantly influenced by ascertainment bias, which was consistent with a previous study in sheep^39^ and cattle^17^.

**Table 3:** Hierarchical analysis of variance based on pairwise differences*.

Source of variance	d.f	Sum of squares	variation components	Percentage of variation	*p* value
Among groups	3	314,971.297	486.39137	1.64	0.029
Among populations within groups	3	234,688.652	1613.69233	5.44	0.00
Among individuals within populations	105	2,870,271.011	− 243.80843	− 0.82	0.645
Within individuals	112	3,116,235.500	27,823.53125	93.75	0.001

*Significance tests based on 1023 permutations.

The Hierarchical F-statistics computed across different groups indicated significant differences in milk production categories (High: dairy breeds; Moderate: Dual breeds; Low: Draft and small statured breeds) and hence differentiated dairy (Sahiwal, Gir and Tharparkar), dual (Ongole, Hariana), and draft (Kangayam) and small sized cattle (Vechur) (Table 4). PCA based on genotype displayed similar categorization of these cattle^17^. The analysis of molecular variance also revealed significant (*p* < 0.05) differences in the proportion of variation (1.64%) due to their functional characteristics viz. dairy, dual, draft and small size. However, there were significant differences (5.44%) between breeds with specific characteristics such as dairy and dual (Table 3). ROH regions observed in these cattle breeds were also able to differentiate dairy and draft breeds as well as small stature cattle^17^.

**Table 4:** Hierarchical F-statistics computed over geographical distribution, body size and level of milk production of 7 cattle breeds.

Hierarchical level	Geographical region	Body size	Level of dairy performance	Individual
Geographical region	0.00	0.19	0.44	0.43
Body size	0.00	0.00	0.30**	0.29
Level of dairy performance (high, medium and low)	0.00	0.00	0.00	− 0.02

**p = 0.001.

### Admixture analyses of the cattle breeds

**Figure 5:**
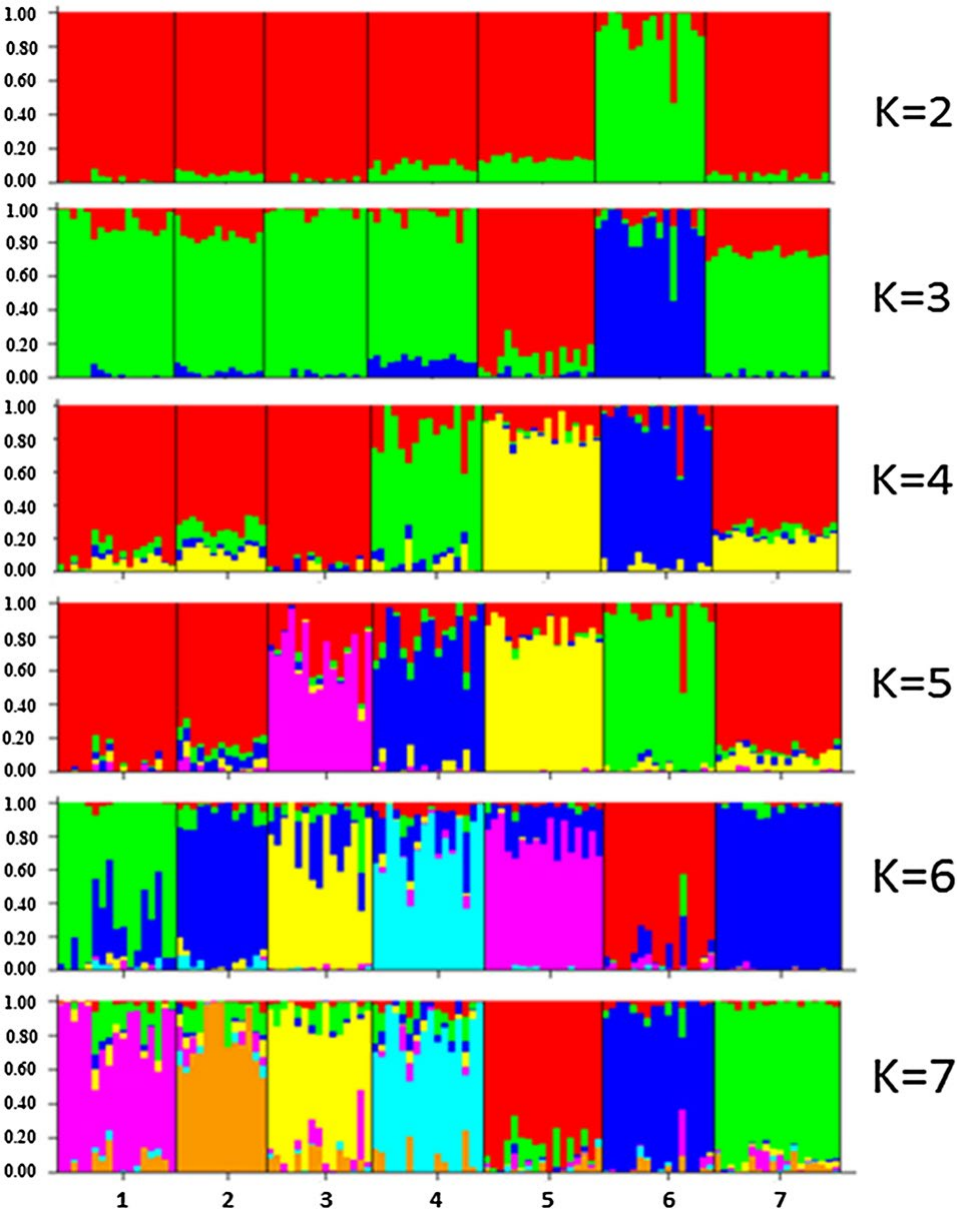
Population structure of seven Indian cattle breeds inferred by using the STRUCTURE software. Each animal is represented by a single vertical line divided into K colors, where K is the number of assumed ancestral clusters, that ranged from 2 to 7, the color segments shows the individual's estimated membership proportions in a given clusters: Breed abbreviations are defined as below:1. Tharparkar, 2. Sahiwal, 3. Gir, 4. Vechur, 5. Ongole, 6. Kangayam, 7. Hariana.

**Figure 6:**
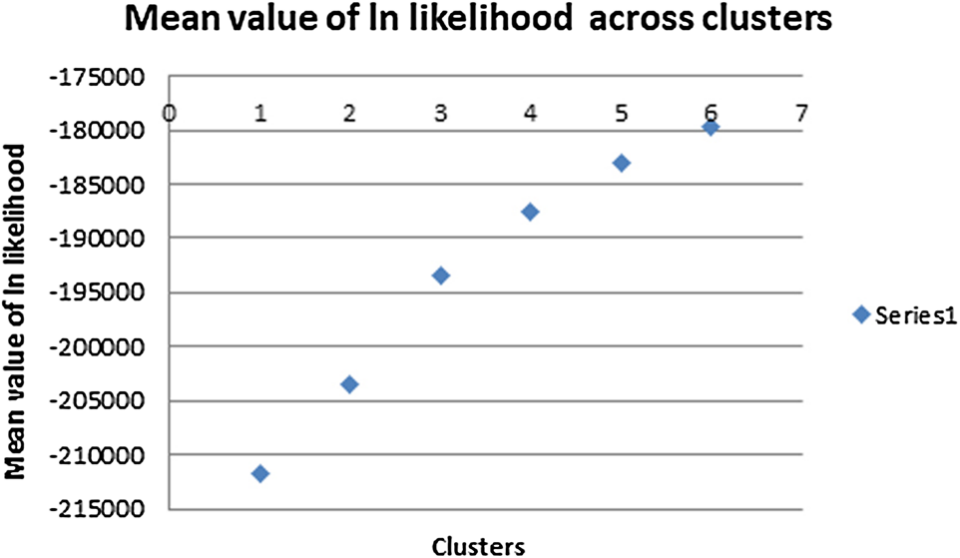
Graphical presentation of mean value of log likelihood across the inferred clusters.

The population structure was studied using admixture model based clustering implemented in Structure^40^ to partition genome of each animal into a predefined number of clusters. The breeds were clearly grouped into draft and dairy/dual breeds (Fig. 5) for pre-defined K = 2, indicating shared ancestry of dairy and dual purpose breeds (Gir, Tharparkar, Sahiwal, Vechur, Ongole, Hariana) very similar to sharing of paternal lineages^41^. More than 90% of genome of dairy /dual cattle except Ongole (86%) clustered together and 90% of the genome of draft cattle Kangayam grouped into separate cluster. At K = 3, Sahiwal, Tharparkar, Gir, Vechur and Hariana (> 72%) clustered together. Kangayam and Ongole clustered into their own group (89%). All major dairy breeds (Tharparkar, Gir and Sahiwal) shared > 80% of their genome. However, at K = 4, Vechur, was clustered apart from other breeds and at K = 5, Gir also clustered separately. At K = 7, most of animals (> 76%) of the different populations under study except Vechur clustered into their own group of animals called breed (Fig. 5). Vechur population displayed highest rate of admixture (Table 5), consistent with its crossing with other breeds and the same was also evident from ROH analysis^17^. Ongole, Kangayam and Hariana shared comparatively less of their genome (≤ 15%) with other breeds. The Fig. 6 revealed the increasing mean value of log likelihood across the inferred clusters and indicated the all seven breeds evolved from their independent ancestry. However, the structure at different values of K showed intermixing of these breeds during the recent past.

**Table 5:** Proportion of membership of each pre-defined population in each of 7 clusters.

Population	Given inferred clusters							Population size
	1 OG	2 HR	3 KG	4 GR	5 TH	6 VC	7 SW	
TH	0.013	0.107	0.014	0.034	0.766	0.016	0.049	17
SW	0.014	0.095	0.014	0.035	0.047	0.026	0.768	13
GR	0.014	0.073	0.014	0.764	0.064	0.011	0.06	15
VC	0.033	0.07	0.052	0.026	0.028	0.733	0.058	16
OG	0.854	0.082	0.01	0.002	0.015	0.011	0.027	17
KG	0.026	0.024	0.889	0.002	0.027	0.01	0.022	16
HR	0.014	0.89	0.005	0.02	0.026	0.01	0.035	18

SW-Sahiwal, TP-Tharparkar, GR-Gir, VC-Vechur, OG-Ongole, KG-Kangayam, HR-Hariana.

### Selection signatures among the breeds

The genomic regions showing most differentiation among breed pairs based on F_ST_ value were identified. The most differentiated breed pairs based on their performance were chosen for the identification of selection signatures. Therefore, Kangayam, a draft breed, was used as control breed for the analysis of dairy breeds while, Vechur, a small sized breed for the rest of large sized breed to search for signatures that may be associated with stature. Genome wide significance level was set to 0.001 to represent a selection signatures. In these selection signatures between distinct breed pairs, 553 genes were found, 40 of which were shared with 412 genes previously identified in these breeds using runs of homozygosity^17^ (Supplementary Table S2, Supplementary Fig. S1). Out of the significantly differentiated genomic regions, the top most differentiating signatures with F_ST_ ≥ 0.50 among different breed pairs is presented in Table 6.

**Table 6:** The selection signature (F_ST_ ≥ 0.50) values among the different cattle breeds and their annotation with Bovine QTL database.

Breed** pair	Top F_ST_ value	Marker	Chromosome	QTLs*
SW/TP	0.67	BovineHD0400009897	BTA4	MY,MC,R,M/C,H,P,M
SW/GR	0.57	BovineHD0500015340	BTA5	MC,R,M/C,H
SW/VC	0.69	BovineHD2900011510	BTA29	ALL TRAITS
SW/HR	0.53	BovineHD0300002536	BTA3	MC,P
SW/KG	0.80	BovineHD2400003366	BTA24	R,M/C,H,M
SW/OG	0.64	BovineHD0800001684	BTA8	R,M/C,P
TP/GR	0.59	BovineHD0300009588	BTA3	ALL TRAITS EXCEPT M
TP/VC	0.73	BovineHD1900008163	BTA19	ALL TRAITS EXCEPT MY
TP/HR	0.50	BovineHD2800011107	BTA28	M/C
TP/KG	0.81	BovineHD1300018382	BTA13	MC,R,M
TP/OG	0.62	BovineHD0200032623	BTA2	ALL TRAITS
GR/VC	0.81	BovineHD1900018844	BTA19	ALL EXCEPT MY
GR/HR	0.60	BovineHD1000011926	BTA10	ALL EXCEPT MY,M
GR/KG	0.81	BovineHD0500019637	BTA5	ALL TRAITS
GR/OG	0.65	BovineHD0500012675	BTA5	ALL TRAITS
VC/HR	0.80	BovineHD1900008163	BTA19	ALL EXCEPT MY
VC/KG	0.76	BovineHD0200022436	BTA2	R,M/C,H,P
VC/OG	0.61	BovineHD1200026864	BTA12	ALL EXCEPT MY
HR/KG	0.80	BovineHD1300013278	BTA13	ALL TRAITS
HR/OG	0.60	BovineHD1700005865	BTA17	ALL EXCEPT MY,H
KG/OG	0.78	BovineHD2100009715	BTA21	R,M/C,H,P

*MY-Milk Yield, MC-Milk Composition, R-Reproduction, M/C-Meat & Carcass.

H-Health, P-Production, M-Morphology.

**SW-Sahiwal, TP-Tharparkar, GR-Gir, VC-Vechur, OG-Ongole, KG-Kangayam, HR-Hariana.

QTLs affecting milk yield, milk composition, reproduction, production, health status, and morphological traits were detected when the top five genomic regions in these cattle were searched in the Bovine QTL database (Supplementary Table S3). The top divergent regions among the most diverse breed pairs may be putative selection signature for differentiating traits between breeds. For example, the most distinguishing genomic region between Kangayam and Sahiwal contains the marker BovineHD0500014902, which is located in the *FAM19A2* gene. This gene is thought to produce brain-specific chemokines or neurokines, which function as immune and nervous cell regulators (https://www.genecards.org/cgi-bin/carddisp.pl?gene=TAFA2), and thus may affect Kangayam and Sahiwal's health in different ways. Kangayam is a hardy draft breed compared to Sahiwal. There were significant differences in number of QTLs affecting milk yield and production traits when Kangayam paired with any of the dairy breed. For example, between Hariana and Kangayam, the marker BovineHD0500012581 lies in *RAB31* gene which is involved in metabolic pathway (https://www.genecards.org/cgi-bin/carddisp.pl?gene=RAB31) and has also been annotated with QTLs affecting milk performance traits (Cattle QTL data base). Hence, may be responsible for variations in these traits.

The other selection signatures identified between Kangayam and dairy breeds included several genes like Bestrophin 3 (*BEST3*) [https://www.genecards.org/cgi-bin/carddisp.pl?gene=BEST3&keywords=BEST3], Diacylglycerol Kinase Alpha (*DGKA*) [https://www.genecards.org/cgi-bin/carddisp.pl?gene=DGKA&keywords=DGKA], Adenosylhomocysteinase (*AHCY*) [https://www.genecards.org/cgi-bin/carddisp.pl?gene=AHCY&keywords=AHCY], Phosphatidylinositol Glycan Anchor Biosynthesis Class U (*PIGU*) [https://www.genecards.org/cgi-bin/carddisp.pl?gene=PIGU&keywords=PIGU] and Phosphofructokinase, Platelet (*PFKP*) [https://www.genecards.org/cgi-bin/carddisp.pl?gene=PFKP&keywords=Phosphofructokinase] which are involved in transportation of glucose and sugars, signaling pathways, cellular processes, cell division and glycolysis regulation, respectively. Moreover, these genomic regions also harbour QTLs affecting milk performance traits (Cattle QTL data base). The signatures were also identified even between the dairy breeds. For example, the genomic region, having BovineHD1300006213 locus, revealing high differentiation between Tharparkar and Gir (F_ST_ = 0.585) harbours QTLs affecting all the traits under study except milk yield (Cattle QTL data). There were significant differences in number of QTLs affecting production (body weight, growth rate etc.) and morphological traits (height etc.) of Vechur when paired with large sized cattle (Supplementary Table S4). The *HMGA2* gene in the genomic region surrounding BovineHD0500013882 locus on chromosome 5 is responsible for explaining the variation in stature of cattle^42^ and human beings^43^. This locus with *HMGA2* gene had high differentiating power (F_ST_ = 0.18) but didn’t appear in the common list of signatures with a threshold value of 0.25 in the present study. Genes such as *FAM19A2*, *BEST3*, *AHCY*, *PIGU*, *PFKP* and *HMGA2* were previously identified while studying runs of homozygosity with the same set of data^17^, thereby validating these signatures.

### Implications of genomic analyses for breed management and conservation

The genomic analyses detailed herein revealed that all of the high yielding dairy breeds, namely Sahiwal, Tharparkar and Gir, shared common ancestry but are admixed to some extent due to gene flow among them through crossing, migration and /or grading up with other breeds. Hariana, traditionally a dual purpose breed found to be closely associated with dairy breeds. In the past, Hariana cattle were subjected to intensive selection for high milk yield and were used as improver breed for many other breeds/populations to augment milk production in the country, and the same was also revealed here through genomic characterization. Ongole, Kangayam and Vechur were quite distinct from rest of the breeds under study. The genomic analyses represented the unique gene pool of these cattle genetic resources, befitting their breed’s status.

In conclusion, BovineHD BeadChip genotyping of Indian cattle is promising for breed structuring, exploring genomic diversity and detecting distinct selection signatures. Hence, it could be used for a wider range of studies, such as genome wide association studies and genomic selection involving larger populations of these breeds. Multi-breed genomic selection may be feasible in dairy breeds due to their shared genome. This study reveals a trend towards shrinking effective population sizes in native Indian cattle breeds, indicating that a long-term breeding strategy is needed to prevent further reductions in Ne, as well as genetic improvement and potential conservation. In future, whole genome sequencing information on these breeds may be useful for pinpointing the genomic regions linked to polygenic productivity, health, fertility, and behavioural traits that evolved under Indian ecological and farming systems.

## Methods

### Animal resources, SNP genotyping and quality control

A total of 132 samples of Sahiwal (SW, n = 19), Tharparkar (TR, n = 17), Gir (GR, n = 16), Ongole (OG, n = 24), Hariana (HR, n = 18), Kangayam (KG, n = 18) and Vechur (VC, n = 20) breeds of cattle were incorporated. Random blood samples were obtained from various farms across the country in accordance with the regulations and guidelines of the Institutional Animal Ethics Committee (IAEC), National Bureau of Animal Genetics Resources (ICAR-NBAGR), Karnal. Genomic DNA was extracted from the whole blood using HiPurATM SPP Blood DNA isolation kit. The quality of the genomic DNA was tested using an agarose gel electrophoresis, and the quantity of DNA was measured using a Nanodrop Spectrophotometer (Nanodrop ND-1000). The DNA samples were genotyped at Sandor Lifesciences Pvt. Ltd. in Hyderabad, India, using an Illumina BovineHD BeadChip with 777,962 SNPs and following the manufacturer's standard procedures. The data files including MAP and PED files were retrieved using Genome Studio. The analysis of the SNP data revealed the call rate ranged from 95 to 99%. The number of SNPs scored in a given sample / the number of SNPs available on chip * 100 was used to calculate the call rate of SNPs. The call rate indicated that the Illumina BovineHD BeadChip is useful in scoring SNP/genotypes in the Indian cattle population and could be used to assess breed signatures and diversity.

The quality control procedure was carried out by using PLINK^44,45^. The unmapped SNPs and SNPs present on X, Y chromosomes, and on mitochondrial DNA were removed and only the SNPs located on autosomes were considered for analysis. SNPs with call rate (CR) ≤ 95%, minor allele frequency (MAF) ≤ 0.05, and HWE (P ≤ 0.001) were excluded. Samples that had more than 10% missing genotypes were also excluded. The quality of SNPs genotyped were assessed based on Gene Call Score (< 0.2) and Gene Train Score (< 0.55) using Genome Studio. The gene train score, in general, was > 0.55 for the SNPs genotyped in these samples and hence good quality SNPs were obtained.

### Genomic diversity analyses among the breeds

For handling and managing as well as analyzing the large size data on 777 K bovine Bead Chip, several in house computer scripts were written for making the suitable data formats for further downstream analyses using different genetic software including HierFstat in R and Structure. LD was measured for each breed as correlation between adjacent SNPs (r^2^) which depends upon the frequencies of the alleles at the loci under consideration. The r^2^ values were calculated using PLINK v 1.9^44,45^ keeping the window size limit of 500 kb between pair-wise SNPs. Further, autosomal SNPs were pruned out with an r^2^ value of 0.5 using PLINK^44,45^. The minor allele frequency, heterozygosity and inbreeding in different breeds were also estimated using PLINK. Hierarchical F-statistics were computed to access genomic differences in different groups using Hierfstat in R (http://www.r-project.org, http://github.com/jgx65/hierfstat): I) geographical distribution (North & South), II) body size (Large & Small), and III) Milk production (High, Medium & Low). The genomic differences among the breeds were also calculated in terms of F_ST_ value^46^ as well as through analysis of molecular variance (AMOVA) using ARLEQUIN^47^.

### Past effective population size (*Ne*)

The historical and recent effective population size (*Ne*) was estimated using the SNeP v1.1 software as described earlier^48^. It inferred Ne based on linkage disequilibrium (LD) against past t generations, where t = 1/2*c* and *c* is the distance between SNPs in Morgans (100 Mb = 1 Morgan was assumed)^49^. The estimation was performed on the SNP data with correction of sample size, phasing and recombination rate.

### Structuring and admixture analyses of the breeds

The population structure was studied using admixture model based clustering implemented in Structure^40^ to partition genome of each animal into a predefined number of clusters (K). The optimum number of K was determined based on mean value of log likelihood across the inferred clusters. The principal component analysis (PCA) based on genomic relationship (IBS-Identity by state) matrix using plink tools and R script was also undertaken to assess breed composition of the animals.

### Detection of diversified selection signatures and the underlying genes

The diversifying selection signatures were identified among the breeds using F-statistics (*P* < 0.0001). F_ST_ value of ≥ 0.25 was considered as selection sweep between two contrasting groups (dairy/dual verses draft, small verses large stature). The highly significant windows between two groups were annotated for their gene content. NCBI map viewer of the bovine UMD3.1.1 (https://www.ncbi.nlm.nih.gov/genome/gdv) was used to identify the genes underlying the selection sweeps. Genes underlying ± 1 MB of SNPs with F_ST_ value of ≥ 0.25 were identified.

The effect of the top 5 signatures on the underlying QTLs was assessed by exploring cattle QTL database (https://www.animalgenome.org/QTLdb/cattle). Test of two proportions was worked out to find the significant differences between the numbers of QTLs affecting the two contrasting groups (dairy versus Draft) for six different traits using XLSTAT.

### Ethics statement

Random blood samples were obtained from various farms across the country with written informed consent from the owner by qualified Veterinarian in accordance with the guidelines issued by the Committee for the Purpose of Control and Supervision of Experiments on Animals (CPCSEA; http://cpcsea.nic.in/WriteReadData/userfiles/file/Compendium%20of%20CPCSEA.pdf) and approved by the Institutional Animal Ethics Committee (IAEC) of ICAR-National Bureau of Animal Genetics Resources (ICAR-NBAGR), Karnal.

## Supplementary Information

Supplementary Information 1.

Supplementary Information 2.

Supplementary Information 3.

Supplementary Information 4.

Supplementary Information 5.

## Data Availability

We have uploaded the data on ICAR-Krishi portal and is in public domain with the URL http://krishi.icar.gov.in/jspui/handle/123456789/31167.
