# Supplementary figures and images for: Genome analyses revealed genetic admixture and selection signatures in Bos indicus
Source: Sci Rep. 2021 Nov 9;11:21924. doi: 10.1038/s41598-021-01144-2 (PMC8578574; doi:10.1038/s41598-021-01144-2)

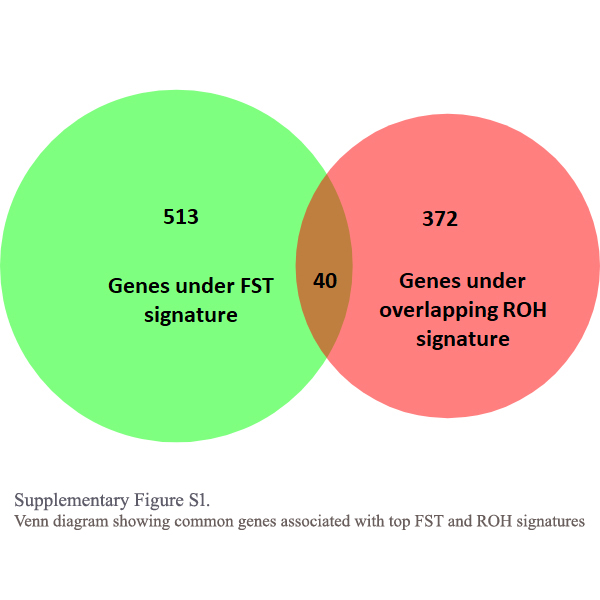

Supplement: Supplementary file 1 — Supplementary Information 1. [file 41598_2021_1144_MOESM1_ESM.jpg]
