# Supplementary material for: Genome analyses revealed genetic admixture and selection signatures in Bos indicus
Source: Sci Rep. 2021 Nov 9;11:21924. doi: 10.1038/s41598-021-01144-2 (PMC8578574; doi:10.1038/s41598-021-01144-2)
Supplement: Supplementary file 2 — Supplementary Information 2. [file 41598_2021_1144_MOESM2_ESM.docx]

**Supplementary Table S1**: The estimated *Ne* in seven Indian native cattle breeds over the past generations

| Generation | KG | HR | GR | OG | SW | TP | VC | All breeds |
| --- | --- | --- | --- | --- | --- | --- | --- | --- |
| 13 | 48 | 74 | 54 | 70 | 51 | 65 | 54 | 373 |
| 15 | 53 | 83 | 61 | 78 | 58 | 73 | 61 | 418 |
| 17 | 60 | 95 | 69 | 88 | 67 | 84 | 70 | 473 |
| 20 | 68 | 108 | 79 | 100 | 76 | 95 | 80 | 543 |
| 23 | 77 | 124 | 91 | 114 | 88 | 110 | 92 | 620 |
| 27 | 89 | 143 | 105 | 130 | 103 | 127 | 107 | 711 |
| 32 | 102 | 167 | 122 | 151 | 120 | 148 | 125 | 821 |
| 38 | 118 | 195 | 144 | 175 | 141 | 172 | 147 | 954 |
| 45 | 138 | 228 | 168 | 204 | 166 | 204 | 173 | 1100 |
| 54 | 163 | 269 | 198 | 240 | 198 | 242 | 206 | 1271 |
| 65 | 191 | 318 | 234 | 282 | 238 | 288 | 245 | 1438 |
| 80 | 227 | 377 | 278 | 331 | 285 | 341 | 292 | 1608 |
| 98 | 270 | 451 | 331 | 391 | 342 | 407 | 350 | 1773 |
| 120 | 324 | 536 | 392 | 461 | 410 | 484 | 419 | 1897 |
| 150 | 384 | 627 | 466 | 539 | 489 | 568 | 499 | 1977 |
| 187 | 456 | 727 | 550 | 630 | 580 | 665 | 591 | 1982 |
| 234 | 542 | 840 | 645 | 729 | 679 | 774 | 692 | 1978 |
| 293 | 644 | 964 | 754 | 839 | 794 | 892 | 806 | 1979 |
| 366 | 757 | 1104 | 875 | 965 | 919 | 1021 | 936 | 2004 |
| 454 | 888 | 1251 | 1013 | 1106 | 1058 | 1165 | 1082 | 2054 |
| 553 | 1024 | 1401 | 1155 | 1257 | 1206 | 1309 | 1232 | 2121 |
| 657 | 1167 | 1560 | 1308 | 1404 | 1351 | 1464 | 1384 | 2221 |
| 759 | 1301 | 1707 | 1446 | 1542 | 1486 | 1606 | 1527 | 2316 |
| 847 | 1412 | 1840 | 1569 | 1671 | 1608 | 1734 | 1649 | 2403 |
| 914 | 1510 | 1935 | 1657 | 1763 | 1693 | 1815 | 1744 | 2480 |
| 958 | 1563 | 2002 | 1716 | 1831 | 1739 | 1871 | 1795 | 2493 |
| 983 | 1602 | 2020 | 1751 | 1861 | 1779 | 1917 | 1833 | 2567 |
| 995 | 1643 | 2065 | 1760 | 1876 | 1823 | 1939 | 1860 | 2577 |
| 999 | 1608 | 2011 | 1740 | 1843 | 1818 | 1882 | 1868 | 2568 |
