# Supplementary material for: Genome analyses revealed genetic admixture and selection signatures in Bos indicus
Source: Sci Rep. 2021 Nov 9;11:21924. doi: 10.1038/s41598-021-01144-2 (PMC8578574; doi:10.1038/s41598-021-01144-2)
Supplement: Supplementary file 5 — Supplementary Information 5. [file 41598_2021_1144_MOESM5_ESM.docx]

**Supplementary Table S4:** The significance (p<0.05) of k proportion test for QTL number influencing different traits between breed pairs

|  |
| --- |

|  | SW/TP | SW/GR | SW/VC | SW/HR | SW/KG | SW/OG | TP/GR | TP/VC | TP/HR | TP/KG | TP/OG |
| --- | --- | --- | --- | --- | --- | --- | --- | --- | --- | --- | --- |
| MILK YIELD - MILK COMPOSITION | * | * | * | * |  |  | * | * |  | * |  |
| MILK YIELD - REPRODUCTION |  |  | * |  |  |  |  | * |  |  |  |
| MILK YIELD - MEAT & CARCASS |  | * |  | * | * |  | * | * |  |  |  |
| MILK YIELD - HEALTH |  |  |  |  |  |  | * |  |  |  |  |
| MILK YIELD - PRODUCTION |  | * | * | * | * | * |  | * |  | * |  |
| MILK YIELD - EXTERIOR MORPHOLOGY |  |  |  |  |  |  |  |  |  |  |  |
| MILK COMPOSITION - REPRODUCTION |  | * |  | * |  |  | * |  |  | * |  |
| MILK COMPOSITION - MEAT & CARCASS |  |  |  |  |  |  |  |  |  |  |  |
| MILK COMPOSITION - HEALTH |  | * |  | * |  |  |  |  |  | * |  |
| MILK COMPOSITION - PRODUCTION |  | * |  | * |  |  | * |  |  |  |  |
| MILK COMPOSITION - EXTERIOR MORPHOLOGY |  | * |  | * |  |  | * | * |  |  |  |
| REPRODUCTION - MEAT & CARCASS |  | * |  | * | * |  | * |  |  |  |  |
| REPRODUCTION - HEALTH |  |  |  |  |  |  |  |  |  |  |  |
| REPRODUCTION - PRODUCTION |  |  |  |  | * |  |  |  |  | * |  |
| REPRODUCTION - EXTERIOR MORPHOLOGY |  |  |  |  |  |  |  | * |  |  |  |
| MEAT & CARCASS - HEALTH |  | * |  | * |  |  |  |  |  |  | * |
| MEAT & CARCASS - PRODUCTION |  |  |  |  |  |  | * |  |  |  |  |
| MEAT & CARCASS - EXTERIOR MORPHOLOGY |  | * |  | * |  |  | * |  |  |  |  |
| HEALTH - PRODUCTION |  | * |  | * |  |  | * |  |  | * | * |
| HEALTH - EXTERIOR MORPHOLOGY |  |  |  |  |  |  | * |  |  |  |  |
| PRODUCTION - EXTERIOR MORPHOLOGY |  |  | * |  |  | * |  | * |  |  |  |
|  |  |  |  |  |  |  |  |  |  |  |  |

|  | GR/VC | GR/HR | GR/KG | GR/OG | VC/HR | VC/KG | VC/OG | HR/KG | HR/OG | KG/OG |
| --- | --- | --- | --- | --- | --- | --- | --- | --- | --- | --- |
| MILK YIELD - MILK COMPOSITION |  |  |  | * |  |  | * | * | * |  |
| MILK YIELD - REPRODUCTION | * |  |  |  |  | * | * |  |  |  |
| MILK YIELD - MEAT & CARCASS | * |  |  | * |  |  |  | * | * |  |
| MILK YIELD - HEALTH |  |  |  |  |  |  |  |  |  |  |
| MILK YIELD - PRODUCTION | * |  | * |  |  |  | * | * |  |  |
| MILK YIELD - EXTERIOR MORPHOLOGY |  |  |  |  |  |  |  |  |  |  |
| MILK COMPOSITION - REPRODUCTION |  |  |  | * |  |  |  | * |  | * |
| MILK COMPOSITION - MEAT & CARCASS |  |  |  |  | * |  |  |  |  | * |
| MILK COMPOSITION - HEALTH |  |  |  | * |  |  |  |  | * | * |
| MILK COMPOSITION - PRODUCTION |  |  |  |  |  |  |  |  |  |  |
| MILK COMPOSITION - EXTERIOR MORPHOLOGY |  |  |  | * |  |  |  |  |  |  |
| REPRODUCTION - MEAT & CARCASS |  |  |  | * |  |  |  | * |  |  |
| REPRODUCTION - HEALTH |  |  |  |  |  | * |  |  |  |  |
| REPRODUCTION - PRODUCTION |  |  | * | * |  |  |  | * |  | * |
| REPRODUCTION - EXTERIOR MORPHOLOGY |  |  |  |  | * |  |  |  |  | * |
| MEAT & CARCASS - HEALTH |  |  |  | * | * |  |  |  | * | * |
| MEAT & CARCASS - PRODUCTION |  |  |  |  | * |  | * |  |  | * |
| MEAT & CARCASS - EXTERIOR MORPHOLOGY |  |  |  | * | * |  |  |  |  | * |
| HEALTH - PRODUCTION |  |  | * |  |  |  | * |  |  |  |
| HEALTH - EXTERIOR MORPHOLOGY |  |  |  |  |  |  |  |  |  |  |
| PRODUCTION - EXTERIOR MORPHOLOGY | * |  | * | * | * |  | * |  |  |  |
|  |  |  |  |  |  |  |  |  |  |  |
